# Rethinking Opsins

**DOI:** 10.1093/molbev/msac033

**Published:** 2022-02-10

**Authors:** Roberto Feuda, Anant K Menon, Martin C Göpfert

**Affiliations:** 1 Department of Genetics and Genome Biology, University of Leicester, Leicester, United Kingdom; 2 Department of Biochemistry, Weill Cornell Medical College, New York, NY, USA; 3 Department of Cellular Neurobiology, University of Göttingen, Göttingen, Germany

**Keywords:** sensory transduction, photosensitivity, vision, moonlighting proteins, G protein-coupled receptors, sensory cell evolution

## Abstract

Opsins, the protein moieties of animal visual photo-pigments, have emerged as moonlighting proteins with diverse, light-dependent and -independent physiological functions. This raises the need to revise some basic assumptions concerning opsin expression, structure, classification, and evolution.

## Introduction: Expanded Functions of Opsins

Opsins are G protein-coupled receptors (GPCRs) that mediate animal vision (Terakita 2005). Their light-sensitivity relies on a covalently bound visual chromophore—usually 11-cis-retinal—that isomerizes upon photon absorption. Besides initiating visual phototransduction, opsins serve nonvisual light-dependent roles in various tissues including the skin and the brain, contributing to processes such as pigmentation changes, avoidance behaviors, hair growth, gonad maturation, and the entrainment of circadian rhythms (Leung and Montell 2017).

Over the past decade, various additional functions have been ascribed to opsins that are light-independent. For example, opsins are expressed in vertebrate taste buds (Sukumaran et al. 2017) and mechanosensory neuromasts of the lateral line (Backfisch et al. 2013; Baker et al. 2015), and they contribute to sperm thermotaxis (Pérez-Cerezales et al. 2015). In the marine bristleworm *Platyneries*, opsins are expressed in cells with both photo- and mechanosensory signatures (Revilla-I-Domingo et al. 2021), whereas in *Drosophila*, opsins are involved in thermal behaviors (Shen et al. 2011), hearing (Senthilan et al. 2012), proprioception (Zanini et al. 2018), and taste sensation (Leung et al. 2020). In *Drosophila* thermoreceptor cells, opsins, though thermostable, might act as thermo-activated receptor proteins (Shen et al. 2011; Leung and Montell 2017), and, in taste transduction, opsins seem to function as chemically activated gustatory receptor proteins (Leung et al. 2020). The latter chemosensory function is independent of chromophore-binding. Instead, bitter compounds can activate opsins, indicating that besides photosensitive chromophores, opsins can have other ligands.

**Fig. 1.**
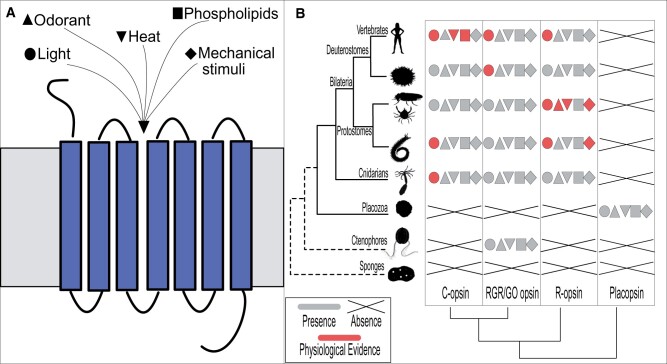
Synopsis of the functional diversity of opsins. (*A*) Schematic representation of functional diversity of opsins. Opsin is depicted generically as a polytopic membrane protein with seven transmembrane spans, in the context of a membrane bilayer. (*B*) Phylogenetic distribution of main metazoan opsin paralogs (according to Feuda et al. [2012]) and their respective functions. The species tree reflects that reported by Feuda et al. (2017), and the dashed lines indicate the uncertainty in the phylogenetic position of ctenophores (Feuda et al. 2017, Li et al. 2021). Symbols as in panel (*A*). Echinoderm R-opsins are expressed in photoreceptors (Ullrich-Lüter et al. 2011), yet physiological evidence implicating them in light detection has not been reported, so the respective symbol is left gray.

In addition to contributing to sensory stimulus transduction, opsins also serve housekeeping functions within cells. For example, unconjugated opsins, without a chromophore, contribute to the maintenance of mechanosensory cilia in *Drosophila* mechanoreceptors; within these cells, opsins localize to the base of the cilia, and, without opsins, these cilia degenerate (Zanini et al. 2018). This opsin-dependence of mechanosensory cilia is remarkable given that it involves nonciliary, rhabdomeric opsins. Opsins can be subdivided into main subfamilies (Terakita 2005; Feuda et al. 2012; Ramirez et al. 2016), that, besides the placozoan-specific placopsins (Feuda et al. 2012), traditionally include the rhabdomeric (R) opsins, the ciliary (C) opsins, and the Go-coupled plus retinochrome, retinal G protein-coupled receptor (Go/RGR) opsins (fig. 1) (for additional discussion, see Ramirez et al. [2016]). Ciliary photoreceptors typically use C opsins, and rhabdomeric photoreceptors R opsins, so the R opsin-dependence of mechanosensory cilia seems unconventional in more than one sense. Opsins can also scramble phospholipids, that is, they act as lipid transport proteins to facilitate rapid, bidirectional movement of lipids from one side of the membrane to the opposing side (Menon et al. 2011), presumably contributing to homeostasis of the disc membranes of mammalian rod photoreceptor cells (Ernst and Menon 2015). The scramblase activity, which opsins share with other rhodopsin-class GPCRs such as the β1- and β2-adrenergic receptors, is particular in that it is ATP-independent and requires neither light nor chromophore-binding (Goren et al. 2014). Finally, opsins regulate melanin levels in skin melanocytes in a light-independent manner (Ozdeslik et al. 2019) and they also serve as trafficking guides for other proteins, physically binding—and acting as a vehicle for—guanylate cyclase 1, the second messenger-generating enzyme of the vertebrate phototransduction cascade (Pearring et al. 2015).

Given these diverse functions, opsins can no longer be considered exclusively as molecular light sensors—they are moonlighting proteins (Jeffery 2014, 2018) that, analogous to moonlighting people, have several, apparently unrelated jobs. Here we argue that this expanded functional diversity challenges some basic assumptions concerning opsins, five of which are spotlighted here.

### Opsin Presence Does Not Necessarily Imply Photosensitivity

Opsin expression alone does not suffice to delineate photosensitive cells. Especially when opsins occur in cells outside eyes, establishing photosensitivity requires physiological studies demonstrating cellular light-responses electrophysiologically or by functional imaging, whereas gene expression and cell morphology can only provide hints. A striking example of this is the expression of opsin genes in sound receptors of the antennal hearing organ of *Drosophila*. Initially, it seemed reasonable to assume that these sound receptors use opsins to detect light—a notion that seems supported by the presence of additional components of the phototransduction machinery in these cells (Senthilan et al. 2012), along with visual cycle proteins (Katana et al. 2019). Multimodal cells that respond to both mechanical stimuli and light occur in *Drosophila* (Xiang et al. 2010), yet the fly’s sound-receptors turned out to be light-unresponsive, requiring opsins to transduce sound stimuli (Senthilan et al. 2012). Genetic manipulations showed that mechanosensory opsin function does not require chromophore-binding (Katana et al. 2019) and that opsins, rather that functioning directly in mechanosensory stimulus transduction, have structural roles, contributing to mechanoreceptor cell integrity (Zanini et al. 2018). Clearly, many cells use opsins for photon-detection, yet there are exceptions that must be kept in mind when inferring cellular functions from the presence of opsins.

### There May Be No Visual and Nonvisual Opsins

Opsins are commonly categorized as visual and nonvisual, depending on whether they contribute to retinal image formation. Functional data now suggest that this dichotomy is far from accurate. In *Drosophila*, opsins that mediate vision also have thermo-, mechano-, and chemosensory roles, documenting nonvisual functions for “visual opsins” (Leung and Montell 2017). *Drosophila* Rh1 for example, the major visual opsin of the fly’s compound eye, contributes to the proprioceptive control of locomotion (Zanini et al. 2018) and the sensation of temperature (Shen et al. 2011) and taste (Leung et al. 2020). This convergence of visual and nonvisual functions on single opsins seems not to be insect-specific. One of the three opsins contributing to human color vision, the short-wavelength-sensitive cone opsin OPN1-SW, occurs in epidermal cells of the skin where it presumably serves nonvisual functions (Haltaufderhyde et al. 2015). The same applies to the human opsin OPN2 that, besides mediating dim-light photoreception in retinal rods, is expressed in the skin, in melano- and keratinocytes (Haltaufderhyde et al. 2015). Even the photoreceptors in the mammalian retina not only serve retinal image formation. In addition, rods and cones contribute to the pupillary reflex and circadian light entrainment, together with retinal ganglion cells that are intrinsically photosensitive, expressing the R opsin melanopsin (OPN4) (Hattar et al. 2002, 2003). Vertebrate melanopsins seems to serve nonvisual functions only, whereas vision in invertebrates such as insects and cephalopods relies on melanopsin-related R opsins (Koyanagi and Terakita 2008). Obviously, the strict dichtonomy between visual and nonvisual opsins does not reflect the complex functions of opsins and their cooption by different types of cells in different organisms. In praxis, opsins serving vision may be referred to as “visual opsins,” yet when doing so one should not forget that additional functions might exist that are equally important for the organisms.

### Opsin Structures Might Reflect Nonlight-Sensing Demands

Like other GPCRs, opsins are heptahelical proteins. Their seventh transmembrane domain (TM7) usually contains a conserved lysine-residue that binds the retinal chromophore via a Schiff-base linkage (Terakita 2005). The structural basis of opsin photo-activation has been studied extensively, with high-resolution structures and biophysical analyses providing detailed insights into light-induced conformational changes (Menon et al. 2001). As opsin function is not restricted to photon-detection, it seems reasonable to assume that additional selective pressures have left their marks on opsin structures. Phospholipid scrambling by bovine opsin, for example, involves conformational changes to TM5-7, which together form a hydrophilic cavity, or groove (Morra et al. 2018; Khelashvili and Menon 2022). With this polar groove, the protein acts like a credit card reader, allowing phospholipids to swipe their way between membrane leaflets with the headgroup moving through the groove, dragging the hydrophobic tails through the membrane core (Pomorski and Menon 2006). Deformations of the membrane that are associated with groove dilation bring together the two bilayer leaflets, additionally facilitating phospholipid flip-flop outside the groove, at the protein-membrane interface (Malvezzi et al. 2018; Khelashvili and Menon 2022). The conformational changes necessary for scrambling are unrelated to those that accompany the photoisomerization of retinal during light transduction (Morra et al. 2018; Khelashvili and Menon 2022). The chemoactivation of opsins represents another pathway for structure evolution unrelated to light-sensing. Certain tastants seem to fit into the chromophore binding pocket of opsins (Leung et al. 2020), suggesting that alternative activation mechanisms and functions must be taken into account when assessing opsin structure-function relations.

### Ancestral Opsin Function Might Not Have Been the Detection of Light

Opsins evolved through duplication in stem eumetazoans, giving rise to melatonin receptors and proto-opsin genes (Feuda et al. 2012).The ancestral function of opsins is often thought to have been the detection of light (light first), even though ancestral opsins such as the placopsins lack the key lysine necessary for chromophore-binding (Feuda et al. 2012). Considering the multiple roles of opsins, it seems possible that light-independent opsin functions evolutionarily preceded light-dependent ones, (nonlight first), or that the first opsins served both light-dependent- and -independent roles (light and nonlight first). Support for a “light first” scenario comes from the extensive association between opsin genes and visual systems, with almost all metazoans using opsins for detecting light. Furthermore, light-independent functions of opsin such as those described in *Drosophila* rely on opsins uniquely present in Brachyceran flies, suggesting that their light-independent sensory functions are derived (Pisani et al. 2020). Residues required for phospholipid scrambling appear to be conserved across GPCRs (Morra et al. 2018; Khelashvili and Menon 2022). Accordingly, opsins presumably inherited their phospholipid scrambling activity from their GPCR ancestor, implying that the first opsins scrambled phospholipids, without (“nonlight first” scenario) or in addition to detecting light (“light and nonlight first” scenario). Even though *Drosophila* opsins are evolutionarily derived, it also seems possible that their light-independent sensory functions have deep evolutionary roots given the presence of opsins in vertebrate taste buds (Haltaufderhyde et al. 2015) and mechanosensory neuromasts (Backfisch et al. 2013; Baker et al. 2015). To discriminate between these three scenarios, more information will be needed about the molecular and physiological functions of opsins outside visual systems, in particular in nonbilaterian metazoans (fig. 1).

### Selection Acting on Light-Independent Functions Might Have Contributed to Opsin Diversification

Since their first appearance, opsins have witnessed an almost bewildering evolutionary diversification, turning into the largest class of GPCRs with more than 1,000 members (Terakita 2005) and with several lineage-specific expansions in some taxa (Davies et al. 2015; Futahashi et al. 2015; Beaudry et al. 2017; Musilova et al. 2019). The main driving force for this diversification is thought to be the demands of light-sensing, in particular color vision (Futahashi et al. 2015; Melin et al. 2017; Musilova et al. 2019; Hauser et al. 2021; van der Kooi et al. 2021); opsins are rendered light-sensitive by chromophore-binding, yet spectral tuning is determined by the opsins themselves (Terakita 2005). Most animals use only two to four opsins for color vision, and a few classes of photoreceptor cells expressing different opsins are sufficient for encoding light in the visible spectrum (Barlow 1982; Cronin et al. 2014). This number contrasts with the large number of opsins that are found in some species (Davies et al. 2015; Futahashi et al. 2015; Beaudry et al. 2017), with the water flea *Daphnia pulex* holding the record of no less than 46 opsins encoded in the genome (Colbourne et al. 2011). The disparity between the numbers of opsins encoded by some genomes and those required for vision indicates that additional factors have contributed to opsin diversification. Clearly, more work is needed to decipher the range of selective pressures contributing to this evolutionary drive and its physiological consequences.
